# A new landscape for malaria vaccine development

**DOI:** 10.1371/journal.ppat.1012309

**Published:** 2024-06-27

**Authors:** Alexander J. Laurenson, Matthew B. Laurens

**Affiliations:** 1 Center for Vaccine Development and Global Health, University of Maryland School of Medicine, Baltimore, Maryland, United States of America; 2 Molecular Microbiology and Immunology Program, Graduate Program in Life Sciences, University of Maryland School of Medicine, Baltimore, Maryland, United States of America; Children’s Hospital of Philadelphia, UNITED STATES

On October 6, 2021, the World Health Organization (WHO) recommended the first vaccine against malaria to prevent *Plasmodium falciparum* malaria in children living in areas with moderate to high transmission [1], a watershed moment in child health. This historic event was informed by results of WHO pilot implementation of the RTS,S vaccination in Ghana, Kenya, and Malawi, that documented feasibility to deliver through routine immunization systems, capacity to increase equity to malaria prevention, a strong safety profile, significant reduction in severe malaria, and high cost effectiveness [2]. More recent analysis of the RTS,S pilot implementation results demonstrated 13% all-cause mortality reduction even in the presence of only moderate vaccine coverage [3]. Enthusiasm for RTS,S implementation in endemic countries has resulted in 18 country approvals to date for Gavi support for vaccine introduction, and current limited supply through 2025 was allocated to 12 of these countries [4].

Two years later, the WHO recommended a second malaria vaccine R21/Matrix-M (R21) on October 2, 2023 [5]. Like RTS,S, R21 generates immunity to *P*. *falciparum* circumsporozoite protein (CSP). A recent Phase 3 clinical trial of R21 in children 5 to 36 months of age demonstrated 75% efficacy at 2 sites with seasonal transmission and 68% efficacy at 3 sites with perennial transmission [6]. While RTS,S and R21 have not been compared head-to-head, they are expected to perform similarly and substantially impact malaria morbidity and mortality in endemic areas. R21 has a significant cost advantage at US $2 to 4 per dose and is expected to fill the huge demand-supply gap.

Now, with 2 high-impact malaria vaccines becoming available, how has this milestone influenced malaria vaccine research and development efforts? This article aims to explain more about the current landscape of malaria vaccine development.

## Question 1. Why are more candidate vaccines needed for malaria?

Although 2 vaccines are recommended, neither meet the desired efficacy and durability for an optimal malaria vaccine. WHO’s preferred product characteristics for a malaria vaccine target a 90% reduction in blood stage infection and clinical malaria over 12 months [7]. When administered seasonally alongside seasonal malaria chemoprophylaxis as a three-dose series, during 12 months of follow-up, RTS,S demonstrated 72% efficacy [8], and R21 demonstrated 75% efficacy [6]. Vaccine-induced immunity wanes over time, which is somewhat mitigated by a fourth and possibly fifth annual booster. Next-generation vaccines that provide even higher efficacy can achieve greater public health impact, possibly requiring fewer doses and no annual booster. Such vaccines could increase individual protection, decrease vaccine delivery system demands, improve cost effectiveness, and further increase equity to malaria prevention.

Similar to COVID-19 vaccine development, multiple vaccine products are needed to ensure vaccine supply. Though not always foreseeable or desirable, any manufacturing or safety concern could surface and indefinitely remove a vaccine from use and necessitate use of an alternate product. Plans to produce malaria vaccines in India and sub-Saharan Africa will increase capacity to meet the current demand. Having multiple products manufactured in different facilities would help to ensure replacement product is available and to provide endemic countries with uninterrupted vaccine access.

## Question 2. What might next-generation malaria vaccines look like?

Many next-generation malaria vaccines are currently in clinical testing (Table 1). Some use novel approaches including live attenuated sporozoite inoculations, RNA-based platforms, and a combination of existing *P*. *falciparum* CSP-based vaccines with antigens from other stages of the parasite life cycle. Live attenuated sporozoite approaches build on human studies that demonstrated 90% protection against malaria infection among adults immunized with radiation-attenuated sporozoites administered via at least 1,000 infected bites [9]. Subsequent advances in cryopreservation of live sporozoites has led to whole organism vaccination regimens tested in the US, Europe, and sub-Saharan Africa, which all demonstrate protection against *P*. *falciparum* malaria [10]. Researchers are now planning trials of late liver stage-arresting, replication competent (LARC), genetically attenuated *P*. *falciparum* sporozoite vaccines that build on safety, immunogenicity, and efficacy demonstrated using previous generation whole sporozoite vaccines but multiply asexually in the liver and thus provide a prolonged stimulation of infection-blocking immune responses.

**Table 1:** Candidate malaria vaccines in clinical testing*.

Antigen(s)	Adjuvant(s)	Phase	Population	Location	ClinicalTrials.gov identifier
***Plasmodium falciparum* Pre-erythrocytic**					
CSP (RNA-based)	-	1	60 adults 15–55 years old	USA sites	NCT05581641
CSP and conserved, immunogenic segments of liver stage-expressed proteins (RNA-based)	-	1/2	160 adults 15–55 years old	USA sites	NCT06069544
CSP (virus-like particle based RTS,S)	AS01E	4	77,000 children up to 5 years old	Ghana, Kenya, Malawi	NCT03855995
CSP (virus-like particle based R21)	Matrix-M	2	330 females of childbearing potential 18–35 years old	Bamako, Mali	NCT06080243
CSP (virus-like particle based R21)	Matrix-M	1	590 children 5–36 months old	Bougouni, Mali	NCT05155579
CSP (virus-like particle based R21)	Matrix-M	1	120 children 5–36 months old living with HIV	Kampala, Wakiso, and Entebbe, Uganda	NCT05385510
CSP (virus-like particle based R21) and ME-TRAP (ChAd63-and-MVA-vectored)	Matrix-M	2	64 adults 18–45 years old	Kilifi, Kenya	NCT03947190
Whole sporozoite (genetically attenuated)	BCG and YF-17D	1	45 adults 18–35 years old	Leiden, the Netherlands	NCT05468606
Whole sporozoite (chemoattenuated) and ME-TRAP (ChAd63-and-MVA-vectored)	-	1/2	30 adults 18–45 years old	Tübingen, Germany	NCT05441410
Whole sporozoite (chemoattenuated and radiation-attenuated)	-	2	372 males 18–55 years old	Jakarta, Indonesia	NCT03503058
Whole sporozoite (radiation-attenuated)	-	2	562 females of childbearing potential 18–38 years old	Ouelessebougou, Mali	NCT03989102
***Plasmodium falciparum* Bloodstage**					
MSP1	GLA-SE	1	40 adults 18–45 years old	Bagamoyo, Tanzania	NCT05644067
MSP3 conjugated to CRM	Alhydrogel	1	42 adults 18–55 years old	Bamako, Mali	NCT05197751
MSP3 conjugated to CRM	Alhydrogel	1/2	465 children 12–59 months old	Bamako, Mali	NCT05776017
RH5.1	Matrix-M	1	24 adults 18–50 years old	Sheffield, United Kingdom	NCT06141057
RH5.1	Matrix-M	1	60 adults 18–45 years old	Bagamoyo, Tanzania	NCT04318002
RH5.1 and RH5.2	Matrix-M	1/2	58 adults 18–45 years old	Oxford, United Kingdom	NCT05978037
RH5.1 and RH5.2	Matrix-M	2	480 children 5–17 months old	Boulkiemdé Province, Burkina Faso	NCT05790889
RH5.1, RIPR, and CyRPA	Matrix-M	1	40 adults 18–45 years old	Oxford, United Kingdom	NCT05385471
***Plasmodium falciparum* Combined life cycle stages**					
RH5.2 (virus-like particle based) and CSP (virus-like particle based R21)	Matrix-M	1	96 participants, including adults 18–45 years old and infants 5–17 months old	Banjul, Gambia	NCT05357560
***Plasmodium falciparum* Transmission-blocking**					
AnAPN1	GLA-LSQ	1	33 adults 18–45 years old	Lambaréné, Gabon	NCT05905432
Pfs25 (complexed with IMX313)	Matrix-M	1	52 adults 18–45 or children 5–12 years old	Bagamoyo, Tanzania	NCT04271306
Pfs48/45	Matrix-M	1	30 adults 18–45 years old	Oxford, United Kingdom	NCT05400746
***Plasmodium vivax***					
PvRII	Matrix-M	2	36 Adults 20–55 years old	Bangkok, Thailand	NCT05380388
Pvs25 (complexed with IMX313)	Matrix M	1	25 adults 18–45 years old	Oxford, United Kingdom	NCT05270265
Pvs230 (conjugated to EPA)	Matrix-M	1	200 adults 18–50 years old	Bethesda, Maryland, USA	NCT05913973

*Clinical trial entries found in clinicaltrials.gov using the search term “malaria vaccine” and restricting to active clinical trials.

**AnAPN1**: Anopheline Alanyl Aminopeptidase N; **BCG**: Bacille Calmette-Guérin; **ChAd63**: Chimpanzee adenovirus 63; **CRM**: cross reacting material from diphtheria toxin mutant; **CSP**: circumsporozoite protein; **CyRPA**: cysteine-rich protective antigen; **EPA**: ExoProtein A from *Pseudomonas aeruginosa*; **GLA-LSQ**: glucopyranosyl lipid adjuvant and saponin QS21; **GLA-SE**: glucopyranosyl lipid adjuvant formulated in a stable oil-in-water nano-emulsion; **IMX313**: hybrid of the oligomerization domain of chicken complement inhibitor C4-binding protein; **MSP3**: Merozoite Surface Protein 3; **ME-TRAP**: multiple epitope thrombospondin-related adhesion protein; **MVA**: modified vaccinia virus Ankara; **Pfs**: *Plasmodium falciparum* surface; **PvRII**: *Plasmodium vivax* Duffy binding protein, region II; **Pvs**: *Plasmodium vivax* surface; **RH5**: reticulocyte-binding protein homolog 5; **VLP**: virus-like particle; **RIPR**: reticulocyte-binding protein homolog 5 interacting protein; **YF-17D**: live-attenuated yellow fever 17D.

Based on the recent success of COVID-19 vaccine development, mRNA-lipid nanoparticle technology is being employed for malaria vaccines in 2 human studies (Table 1). mRNA-based vaccines provide advantage as they can be manufactured quickly, are safe and effective for young infants and pregnant women, and can code for multiple antigens to strengthen the immune response. Disadvantages include side effects, though these are generally mild and temporary. The first mRNA-based malaria clinical trial tests a single RNA construct encoding part of the *P*. *falciparum* circumsporozoite protein (CSP), and the second tests a combination of 3 distinct RNAs—the full *P*. *falciparum* CSP and 2 conserved segments of liver stage-expressed proteins—with plans for controlled human malaria infection to determine preliminary vaccine efficacy. Other promising RNA-based malaria vaccine strategies are in preclinical development [11–14].

Another strategy for malaria vaccines focuses on improving RTS,S and R21 efficacy in preventing disease by adding a separate vaccine antigen targeting the parasite’s erythrocytic cycle so a single product would provide both pre-erythrocytic liver stage protection and erythrocytic efficacy against parasitic escape. One such strategy combining R21 with the blood stage antigen reticulocyte-binding protein homolog 5 (RH5) is already underway [15].

## Question 3. How will computational biology inform next-generation malaria vaccines?

Most current malaria vaccine target antigens were discovered by identifying immune responses in following malaria infection, yet few have demonstrated efficacy in clinical studies. Reasons for vaccine failure include antigenic variation, off-target antibody responses diluting intended protective responses, and short durability of immunity [4]. Novel bioinformatics tools can overcome these obstacles by leveraging parasite and human genomic data to strategically identify candidate vaccine targets that generate precise and accurate immunity, and to overcome parasite diversity.

To optimize immunogenicity and targeted immunity, computational techniques such as 3D protein modeling can predict conformation-dependent immune responses to malaria proteins, which allows researchers to identify parasite gene loci that are susceptible to immune escape from vaccine-induced protection [16]. In addition, integrating known local HLA polymorphism and parasite population sequence data from endemic regions to identify T cell epitopes recognizable by common HLA alleles optimizes vaccine design, ensuring results are directly applicable to target populations.

Despite *P*. *falciparum*’s enormous antigenic diversity, comprehensive analyses of parasite genomic and transcriptomic data collected in endemic areas can identify genomic regions under positive selection pressure to remain conserved [17]. These antigens serve as ideal candidate vaccines. Moreover, parasite transcriptomic profile analysis pinpoints essential proteins consistently expressed during distinct life cycle stages that can also serve as vaccine targets [17]. Advanced characterization of *P*. *falciparum*’s complex genome using a combined set of approaches can provide a more credible and well-informed selection of target regions as candidate vaccine antigens for development.

A pipeline approach that incorporates high-throughput analyses in sequence can predict conserved and positively selected antigenic regions that elicit successful and protective immune responses, circumventing traditional preclinical experimentation that is costly and time-consuming. With experimentally validated bioinformatic predictive tools informed by genomic datasets, resources are deployed precisely and efficiently, thus accelerating antigen discovery for preclinical testing.

## Question 4. How will next-generation malaria vaccines be down-selected?

RTS,S underwent a lengthy 35-year development from creation in 1987 [18] to 2021 when the WHO recommended it for use [1]. CSP was identified as a target of the immune response generated by radiation-attenuated sporozoites, and epitope mapping led to development of a subunit vaccine that demonstrated protection against Controlled Human Malaria Infection (CHMI). RTS,S was then tested with multiple adjuvants, in rhesus and then in human clinical trials with CHMI in malaria-naïve adults and subsequently in malaria-exposed adults and then children and infants living in endemic areas [18]. As no known correlate of RTS,S-induced protection against *P*. *falciparum* was identified, efficacy studies in the target population of children living in endemic areas were required to assess RTS,S impact.

Now, with data from multiple clinical trials of RTS,S, recent advances in our understanding of vaccine-induced immunity to *P*. *falciparum* malaria, and refinement of preclinical models, it is possible to use mouse models to improve existing CSP-based vaccines [19]. Adjuvants can now be carefully selected based on the desired effector function, [20] obviating the need for large CHMI and/or efficacy studies to optimize adjuvant selection. Cryo-electron microscopy has advanced understanding of CSP-based structures underlying high antibody avidity and potency needed for an effective vaccine [21]. As regulatory bodies and experienced clinical trial centers exist in malaria endemic areas, candidate next-generation vaccines ready for human testing can be trialed in first-in-human studies with CHMI in endemic countries, lessening the need for initial testing in the US and Europe and potentially shortening time needed for clinical development. Overall, these advances will facilitate efficient testing of improved CSP-based vaccines.

## Question 5. What about vaccines that block transmission?

Vaccines that prevent malaria transmission are needed to achieve elimination goals. A highly effective pre-erythrocytic vaccine would completely prevent parasite erythrocytic development and thus halt onward transmission, though developing a vaccine with 100% efficacy may not be feasible. RTS,S and R21 are pre-erythrocytic vaccines that incompletely prevent blood stage infection, thus improving malaria morbidity and mortality. These vaccines address the first 2 WHO strategic priorities for malaria vaccines to prevent human blood-stage infection at the individual level and to reduce morbidity and mortality in individuals at risk in malaria-endemic areas [7]. However, they do not address the third WHO strategic priority to reduce parasite transmission and incidence of human infection in the community [22]. Malaria vaccines that reduce transmission exclusively would not provide health benefit to an individual but would significantly impact malaria elimination efforts at the community and regional levels.

Vaccines targeting *P*. *falciparum* antigens expressed during parasite sexual development in the mosquito midgut represent a promising approach to prevent malaria transmission to mosquitoes, blocking onward transmission to humans. As these antigens are not seen by the human immune system during parasite development, they are not targets of naturally acquired immunity. Transmission-blocking vaccines can induce antibodies that are subsequently ingested by the mosquito vector during a blood meal and that act directly on parasites. Such vaccines are based on parasite antigens expressed in the mosquito midgut, including Pfs230 and Pfs25 [23], and Pfs48/45 [24]. Transmission-blocking vaccines could be administered as a standalone product or combined with a pre-erythrocytic or erythrocytic vaccine to provide both individual and community benefit.

As clinical trials of transmission-blocking vaccines that measure community transmission as an outcome would require a large number of participants exposed to an investigational product to measure efficacy, immunogenicity studies can be used as proxies. In addition to measurements of antibody against the vaccine antigen, serum functional activity against parasite sexual stage development is measured using a standard membrane feeding assay, where mosquitoes feed on cultured gametocytes in the presence of serum and are then observed for parasite oocyst development within each mosquito [25]. Direct skin feeding assays can also be used where female *Anopheles* are placed in a mesh container and allowed to feed directly at the skin surface of a vaccinated participant, then later dissected to assess for parasite oocyst development [23]. Results of these functional assays inform clinical development, though no transmission-blocking vaccine has progressed beyond Phase 2 testing to date.

## Conclusions

The first 2 malaria vaccines recommended by the WHO in 2021 and 2023 may have arrived just in time, as current malaria case counts remain essentially unchanged since 2015, reports of first-line antimalarial resistance are becoming more common, and climate change threatens recent advances in malaria control. The advent of these vaccines has been met with strong public interest in vaccination as a means to tackle malaria, and signals that future improvements in malaria vaccines will likely achieve similar high demand and uptake. Next-generation vaccines are needed to provide enhanced and sustained efficacy that will improve child health, increase educational outcomes for children, save lives, and advance elimination efforts. Preclinical work to define new and improved vaccine antigens can be informed by computational biology pipelines to increase efficiency. While multiple interventions are needed to control malaria in endemic areas, high-impact interventions that prevent the most illnesses and deaths with available resources are a priority. Malaria vaccines represent a high-impact intervention that can reduce clinical disease, prevent severe malaria illness, decrease hospitalizations, and improve child survival [3]. Vaccines epitomize a viable strategy that can be furthered and advanced through continued research and innovation to accelerate malaria elimination efforts and shrink existing health disparities in resource-limited areas, paving the way toward a malaria-free future.

## References

[1] WHO recommends groundbreaking malaria vaccine for children at risk. World Health Organization; 2021 Oct.

[2] Malaria Vaccine Implementation Programme (MVIP) Programme Advisory Group (PAG). Full Evidence Report on the RTS,S/AS01 Malaria Vaccine. SAGE Yellow Book. 2021 Oct.

[3] Moorthy V, Hamel MJ, Smith PG. Malaria vaccines for children: and now there are two. Lancet. 2024 Feb;403(10426):504–5. doi: 10.1016/S0140-6736(23)02743-5 38310911

[4] Global Malaria Programme (GMP), World Health Organization. World Malaria Report 2023. 2023.

[5] WHO recommends R21/Matrix-M vaccine for malaria prevention in updated advice on immunization. World Health Organization; 2023 Oct.

[6] Datoo MS, Dicko A, Tinto H, Ouédraogo JB, Hamaluba M, Olotu A, et al. Safety and efficacy of malaria vaccine candidate R21/Matrix-M in African children: a multicentre, double-blind, randomised, phase 3 trial. Lancet. 2024 Feb;403(10426):533–44. doi: 10.1016/S0140-6736(23)02511-4 38310910 PMC7618965

[7] Malaria vaccines: preferred product characteristics and clinical development considerations. World Health Organization; 2022 Sep.

[8] Chandramohan D, Zongo I, Sagara I, Cairns M, Yerbanga RS, Diarra M, et al. Seasonal Malaria Vaccination with or without Seasonal Malaria Chemoprevention. N Engl J Med. 2021 Sep 9;385(11):1005–17. doi: 10.1056/NEJMoa2026330 34432975

[9] Luke TC, Hoffman SL. Rationale and plans for developing a non-replicating, metabolically active, radiation-attenuated *Plasmodium falciparum* sporozoite vaccine. J Exp Biol. 2003 Nov 1;206(21):3803–8. 14506215 10.1242/jeb.00644

[10] Richie TL, Church LWP, Murshedkar T, Billingsley PF, James ER, Chen MC, et al. Sporozoite immunization: innovative translational science to support the fight against malaria. Expert Rev Vaccines. 2023 Dec 31;22(1):964–1007. 37571809 10.1080/14760584.2023.2245890 PMC10949369

[11] Mallory KL, Taylor JA, Zou X, Waghela IN, Schneider CG, Sibilo MQ, et al. Messenger RNA expressing PfCSP induces functional, protective immune responses against malaria in mice. NPJ Vaccines. 2021 Jun 18;6(1):84. doi: 10.1038/s41541-021-00345-0 34145286 PMC8213722

[12] Hayashi CTH, Cao Y, Clark LC, Tripathi AK, Zavala F, Dwivedi G, et al. mRNA-LNP expressing PfCSP and Pfs25 vaccine candidates targeting infection and transmission of Plasmodium falciparum. NPJ Vaccines. 2022 Dec 1;7(1):155. doi: 10.1038/s41541-022-00577-8 36456563 PMC9715627

[13] Ganley M, Holz LE, Minnell JJ, De Menezes MN, Burn OK, Poa KCY, et al. mRNA vaccine against malaria tailored for liver-resident memory T cells. Nat Immunol. 2023 Sep;24(9):1487–98. doi: 10.1038/s41590-023-01562-6 37474653

[14] Scaria PV, Roth N, Schwendt K, Muratova OV, Alani N, Lambert LE, et al. mRNA vaccines expressing malaria transmission-blocking antigens Pfs25 and Pfs230D1 induce a functional immune response. NPJ Vaccines. 2024 Jan 6;9(1):9. doi: 10.1038/s41541-023-00783-y 38184666 PMC10771442

[15] University of Oxford. A Study to Assess the Experimental Malaria Vaccines RH5.2-VLP and R21 [Internet]. 2023 Jul. Report No.: NCT05357560. Available from: https://clinicaltrials.gov/study/NCT05357560.

[16] Yurina V, Adianingsih OR. Predicting epitopes for vaccine development using bioinformatics tools. Ther Adv Vaccines Immunother. 2022 Jan;10:251513552211002. doi: 10.1177/25151355221100218 35647486 PMC9130818

[17] Neafsey DE, Taylor AR, MacInnis BL. Advances and opportunities in malaria population genomics. Nat Rev Genet. 2021 Aug;22(8):502–17. doi: 10.1038/s41576-021-00349-5 33833443 PMC8028584

[18] Laurens MB. RTS,S/AS01 vaccine (Mosquirix): an overview. Hum Vaccines Immunother. 2020 Mar 3;16(3):480–9. 10.1080/21645515.2019.1669415 PMC7227679 31545128

[19] Genito CJ, Brooks K, Smith A, Ryan E, Soto K, Li Y, et al. Protective antibody threshold of RTS,S/AS01 malaria vaccine correlates antigen and adjuvant dose in mouse model. NPJ Vaccines. 2023 Aug 10;8(1):114. doi: 10.1038/s41541-023-00714-x 37563255 PMC10415390

[20] Loos C, Coccia M, Didierlaurent AM, Essaghir A, Fallon JK, Lauffenburger D, et al. Systems serology-based comparison of antibody effector functions induced by adjuvanted vaccines to guide vaccine design. NPJ Vaccines. 2023 Mar 8;8(1):34. doi: 10.1038/s41541-023-00613-1 36890168 PMC9992919

[21] Martin GM, Torres JL, Pholcharee T, Oyen D, Flores-Garcia Y, Gibson G, et al. Affinity-matured homotypic interactions induce spectrum of PfCSP structures that influence protection from malaria infection. Nat Commun. 2023 Jul 28;14(1):4546. doi: 10.1038/s41467-023-40151-x 37507365 PMC10382551

[22] Malaria vaccines. World Health Organization; 2024. (Immunization, Vaccines and Biologicals).

[23] Sagara I, Healy SA, Assadou MH, Kone M, Swihart BJ, Kwan JL, et al. Malaria transmission-blocking vaccines Pfs230D1-EPA and Pfs25-EPA in Alhydrogel in healthy Malian adults; a phase 1, randomised, controlled trial. Lancet Infect Dis. 2023 Nov;23(11):1266–79. doi: 10.1016/S1473-3099(23)00276-1 37499679 PMC10615700

[24] Tiono BA, Plieskatt JL, Ouedraogo A, Soulama BI, Miura K, Bougouma EC, et al. A randomized first-in-human Phase 1 trial of differentially adjuvanted Pfs48/45 malaria vaccines in Burkinabé adults. J Clin Invest [Internet]. 2024 Jan 30 [cited 2024 Feb 9]; Available from: http://www.jci.org/articles/view/175707. 10.1172/JCI175707 PMC10977980 38290009

[25] Wu Y, Ellis RD, Shaffer D, Fontes E, Malkin EM, Mahanty S, et al. Phase 1 Trial of Malaria Transmission Blocking Vaccine Candidates Pfs25 and Pvs25 Formulated with Montanide ISA 51. Ratner AJ, editor. PLoS ONE. 2008 Jul 9;3(7):e2636. doi: 10.1371/journal.pone.0002636 18612426 PMC2440546

